# Redox Potential (E_0_′) of the β-Chain 93Cys of HbS Measured with the Equilibrium Technique in a Heterozygous Sickle Cell Carrier Subject

**DOI:** 10.3390/molecules30224342

**Published:** 2025-11-10

**Authors:** Federico Maria Rubino, Aldijana Sadikovic, Camillo Morano, Michele Dei Cas, Monica Bignotto, Sara Ottolenghi, Michele Mondoni, Davide Chiumello, Michele Samaja, Rita Paroni

**Affiliations:** 1 Department of Health Sciences, University of Milano, v. A. di Rudinì 8, 20142 Milan, Italy; aldijana.sadikovic@unimi.it (A.S.); camillo.morano@unimi.it (C.M.); michele.deicas@unimi.it (M.D.C.); monica.bignotto@unimi.it (M.B.); michele.samaja@unimi.it (M.S.); rita.paroni@unimi.it (R.P.); 2 Department of Medicine and Surgery, University of Milano Bicocca, 20126 Milan, Italy; s.ottolenghi@campus.unimib.it; 3 Department of Emergency, Ospedale San Carlo Borromeo, ASST Santi Paolo e Carlo, 20172 Milan, Italy; 4 Respiratory Unit, Department of Health Sciences, ASST Santi Paolo e Carlo, Università degli Studi di Milano, 20145 Milan, Italy; michele.mondoni@unimi.it; 5 Complex Structure of Anesthesia and Intensive Care, Department of Health Sciences, ASST Santi Paolo e Carlo, Università degli Studi di Milano, 20145 Milan, Italy; davide.chiumello@unimi.it

**Keywords:** bio-electrochemistry, erythrocyte, glutathione, glutathione disulphide, hemoglobin A, hemoglobin S, MALDI mass spectrometry, oxidative stress, redox potential, sickle cell disease

## Abstract

The hitherto unknown thiol-disulfide redox potential (E_0_′) of the β93Cys residue in the HbS (β^6Glu→Val^) variant of human hemoglobin was calculated by MALDI-ToF mass spectrometry, which analyzes blood from a heterozygous carrier. To calculate the (E_0_′) value, a redox equilibrium model was adopted, and the previously calculated value for wild-type β-Hb chain (E_0_′ −121 mV) was used. An E_0_′ value of −130.5 ± 1.7 mV for the β93Cys residue of HbS was obtained, thus a more reducing value than E_0_′ in the wild-type isoform. Glutathionylation from this residue in the HbS tetramer lowers the extent of protein aggregation in fibrils and the clinical consequences, such as painful capillary occlusion and hemolysis. This finding confirmed the peculiar property of HbS as a more reactive scavenger of glutathione sulphinic acid (E_0_′ = −264 mV), which forms in the cytoplasm of red blood cells and reacts with structural and regulatory proteins, including hemoglobin. The ability to assess the erythrocyte oxidative status in sickle cell carriers can be developed into an additional functional test to rationally assess the effect of drug treatment and antioxidant dietary interventions on improving disease control.

## 1. Introduction

The sickle cell trait of red blood cells (RBCs) is the first molecular disease for which the genesis has been determined, starting with a DNA single-point mutation (C**T**C > C**A**C) in the β-globin gene and determining a single acid-to-neutral amino acid change, Glu→Val, at position 6, close to the *N*-terminal of a 150-amino-acid-long protein [1,2,3].

The resulting HbS-variant hemoglobin tetramer carries two β^6Glu→Val^-variant chains and has different and unfavorable properties. HbS polymerization under deoxygenated conditions leads to the formation of fibrous precipitates within red blood cells, resulting in altered deformability and impaired oxygen delivery to tissues. This polymerization process, rather than a direct defect in oxygen binding, underlies the pathophysiology of sickle cell disease, including reduced resistance to physical effort and infections, in children. The β^6Glu→Val^ α_2_β_2_ tetramers bind together through non-covalent, hydrophobic forces of the modified conformations [4,5] and generate fibrils that modify the natural discoidal, bi-concave shape of the healthy RBCs and their capacity to deform reversibly to enter the narrowest capillaries of the bone, joints, and internal organs. Irreversibly sickled RBCs cause, as the most critical clinical feature, capillary obstruction, bone pain, and painful swelling of joints due to thrombotic occlusion of micro-vessels, hemolysis, and stroke [6,7]. Sickle cell disease (SCD) affects about 7.7 million people worldwide, and a very recent editorial in *Lancet Hematology* highlights this as a neglected health priority for which more research and funding is needed [8].

Systemic and cellular oxidative stress occurs as the physiological commitment of the organism to variations in the use of oxygen and produces reactive species through a host of different chemical and biological mechanisms. The biochemical antioxidant response of the organism at systemic and intracellular levels is to deactivate reactive species, especially by means of soluble molecular antioxidants and enzyme-catalyzed reactions, before such species can cause chemical and functional damage to bio-structures, such as cellular membranes, structural and functional proteins, enzymes, and DNA. Oxidative stress is either a physiological coping and signaling mechanism, in transient increased oxygen consumption, or a pathological event, when the organism is chronically exposed to low oxygenation and cannot express sufficient coping activity [9,10,11].

The cysteine-containing non-ribosomal tripeptide glutathione (GSH) is a key component of the physiological antioxidant and toxicant response intracellular panel. Most of its antioxidant activity is carried through the reversible formal interconversion of the thiol(ate) functional group of cysteine to the disulfide form. It is worth noting that recently, a non-covalent binding of GSH to hemoglobin has been highlighted [12,13].

The process of reversible hemoglobin glutathionylation is a coping response of RBCs to oxidative stress from endogenous processes that generate hydrogen peroxide [14] and from other substances, such as hydroperoxides [15], peroxynitrite [16], and nitrite [17,18]. It uses the two most abundant buffering species, hemoglobin, at 5 mM typical concentration, and glutathione, at 0.5 to 3.5 mM, as genetically controlled levels [19]. Glutathionyl–hemoglobin (Hb-SSG) is continuously generated and reduced [20] as the key component of the intracellular antioxidant network [21,22]. Further details are in the Supplementary Materials.

Within the complex network of metabolic reactions in RBCs [23,24,25,26], the overall reversible disulfide–thiolate redox reactions constitute a prominent antioxidant buffer that protects more sensitive biological components and structures from the degrading effect of reactive electrophiles and oxygen-derived radical species.

Dynamic conformational studies show that glutathionylation of the reactive-thiol 93Cys residue is even enhanced in the β^6Glu→Val^ Hb variant, compared to that of the wild-type protein [27]. In addition, glutathionylation weakens the intermolecular interaction necessary for association with fibrils [28], thus lowering hemoglobin polymerization and improving RBC blood cell rheology and the symptoms of the disease.

**Figure 1:**
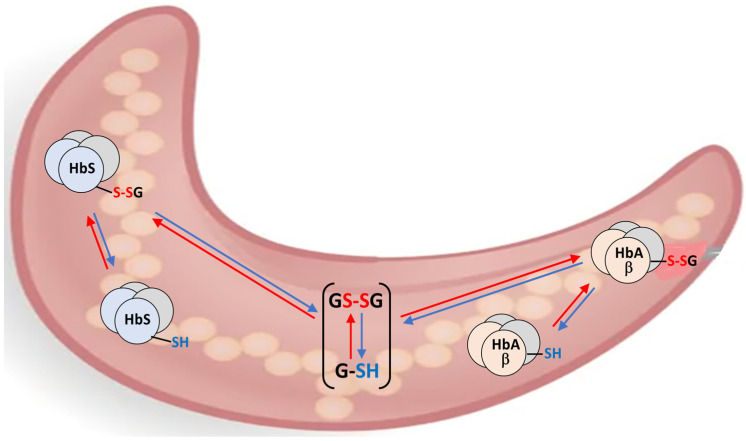
Interplay of three intra-erythrocyte processes of reversible glutathionylation in the sickle RBCs of a heterozygous HbS-variant carrier.

The response of the thiolome of RBCs to oxidative stress involves the reversible distribution of the glutathione pool among its soluble thiol (GSH), disulfide (GSSG), and hemoglobin-bound forms (Hb-SSG), as illustrated in the scheme of Figure 1 for the interplay of the glutathionylated forms of Hemoglobin A and S (HbA-S-SG, HbS-S-SG) in sickled RBCs.

Therefore, the availability of a biochemical parameter able to assess the physiological response of RBCs to conditions that enhance oxidative stress would simplify understanding this complicated network of processes. The values of E_0_′ have been measured for the main components of the soluble thiolome utilizing solution and spectroscopic techniques [29] and by extrapolation of gas-phase behavior to the solution state [30]. In addition, re-analysis [30] of measured concentrations of GSH, GSSG, and Hb-SSG in an oxidant challenge ex vivo, in vitro experiment [31] allowed calculation of the value of E_0_′ for the 93Cys thiol group of wild-type Hb.

The availability of blood from a carrier of the sickle cell β^6Glu→Val^ variant of human β-Hb prompted an analysis of the composition of hemoglobin isoforms and main modifications obtained by MALDI-ToF mass spectrometry to apply a previously developed calculation for obtaining a value for the hitherto unknown thiol-disulfide redox potential (E_0_′) of the 93Cys residue in the modified protein. Availability of this value, in turn, assists in understanding the potentiality of the “redox potential” approach to study RBC physiopathology in conditions of chronic and transient oxidative stress.

## 2. Results

### 2.1. MALDI Mass Spectra of the Patient’s Hemolysates

**Figure 2:**
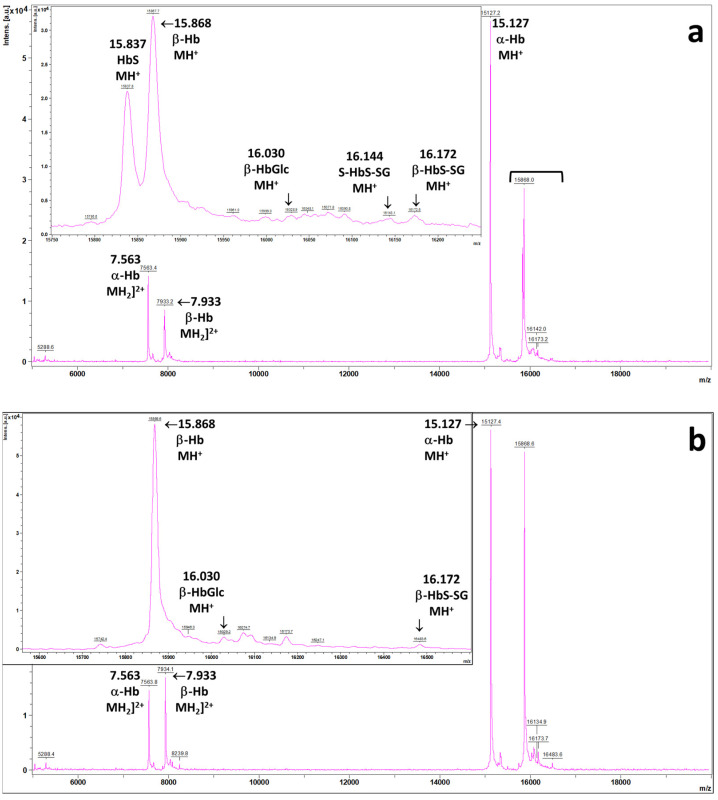
MALDI mass spectra of the hemolysate from (**a**) the profile of the subject carrying the β^6Glu→Val^ variant of human Hb; (**b**) the wild-type hemoglobin subject (main analytical conditions are described in the Materials and Methods section). The expansions highlight the minor forms of the β-chain of the wild type (glycated and glutathonylated, in both panels) and, in panel (**a**), the unmodified and glutathionylated forms of the β^6Glu→Val^-variant chain.

As part of a different research protocol that included assessing HbSSG in the blood of patients [32], one subject used the anomalous MALDI mass spectrum reported in Figure 2a. Apart from minor differences in the relative proportions of the minor signals (*vide infra*), samples from the same patient (n = 2), taken at admission to and discharge from the hospital, showed very similar spectral profiles. As a reference, the spectrum from a wild-type hemoglobin subject is reported as Figure 2b.

The proteins in the samples were as summarized in Table A1. Briefly, the patient’s RBCs contain both the α-(*m*/*z* 15,127) and β-(*m*/*z* 15,868) wild-type hemoglobin chains and, unexpectedly at the time of analysis, an additional signal (*m*/*z* 15,837) that corresponds to the β^6Glu→Val^ variant of the β-chain [33]. The intensity ratio of the two forms of hemoglobin is approximately 61% (β-Hb):39% β^6Glu→Val^), calculated as the average of four replicated sample depositions for each of the two obtained blood samples, as reported in Table A2.

We should raise awareness that relative MALDI intensities of different proteins may not accurately reflect their actual concentration ratios, due to possible differences in their overall ionization and desorption efficiency during the analysis. This uncontrolled factor may influence the calculation of the actual intra-erythrocyte concentrations of the two hemoglobin isoforms, with a likely minor influence also on the ratio of the glutathionylated to free-cysteine form of each. Nevertheless, due to the current lack of suitable standards, the response factors of the two isoforms are taken as identical for the purpose of the ensuing calculations.

The measured 3:2 proportion of the β-Hb and β^6Glu→Val^ Hb chains points to a heterozygous carrier of the sickle cell trait. In addition, the spectrum does not show substantial levels of the micro-heterogeneous fetal γ forms centered at *m*/*z* 15,995 (isoform 137-G) and 16,009 (isoform 137-A), respectively [34,35]. These proteins would be present in large amounts in the RBCs of sickle cell patients treated with hydroxyurea, a drug that stimulates, in the still immature RBCs, the biosynthesis of the fetal chains [36], as we evidenced previously by analyzing an unrelated sample of a homozygous-variant carrier subject undergoing the pharmacological treatment with hydroxyurea.

In addition, as is apparent from the inserts of Figure 2a,b, further minor peaks are present, corresponding to the known minor forms of hemoglobin: the glycated α- and β-forms (*m*/*z* 15,289 and 16,030, respectively) and the glutathionylated β-chain (*m*/*z* 16,172). One further signal, at *m*/*z* 16,142, corresponds to the glutathionylated form of β^6Glu→Val^ chain [36]. Table A3 reports the mean % proportions of the isoforms in the samples.

### 2.2. Calculation of the Value of E_0_ for the 93Cys of HbS

**Figure 3:**
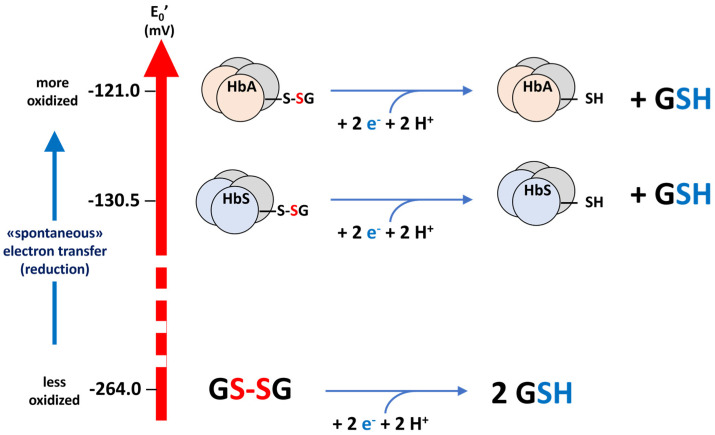
E_0_′ potentials of the redox pairs of glutathione (GSSG/GSH) and of the 93Cys residue in HbS and HbA.

A value for the redox potential of the thiol group in the side chain of 93Cys in β^6Glu→Val^ in HbS could be calculated from the MALDI-ToF mass spectra of the two samples, using the equilibrium method that was developed to calculate the corresponding value for the wild-type protein [31]. The complete calculation is reported in Appendix B. The values of hematocrit and total Hb concentration were used to calculate the total erythrocyte Hb concentration (line 3 of Table A4). In this case, the two forms of hemoglobin compete for the glutathione pool, and their concentrations were calculated from the total Hb content, while the relative proportions of the different forms were measured by mass spectrometry. Then, the calculated concentrations of the different Hb forms are used in the Nernst equation to calculate the value of E_0_′(β^6Glu→Val^-Hb), using the already available E_0_′(β-Hb) [31] as reference. The calculated value of E_0_′(β^6Glu→Val^-Hb) of HbS is −130.5 ± 1.7 mV, as the mean of the eight determinations performed on the two samples. This value corresponds to a more negative potential, and therefore to a more nucleophilic (acid) 93Cys thiolate residue, which is thus more reactive towards thiolate exchange with GSSG (more oxidizable) and less reducible by GSH than the homologous one in the wild-type β chain of HbA. The three processes are shown in the scheme of Figure 3.

### 2.3. Application: The Oxidative Stress Level of the Patient Compared to That of In Vitro Oxidatively Challenged Wild-Type Healthy RBCs

According to the estimated E_0_′ values for the two hemoglobin variants, in the two blood samples of the patient, the calculated concentration-dependent values of the E_h_′ potential, considered at equilibrium, were −172.8 ± 0.1 mV at hospital admission and −175.7 ± 0.2 mV at discharge. A preliminary investigation of the meaning of this observation is developed below.

To frame the condition of chronic oxidative stress of the patient’s sickle RBCs, it is necessary to compare the values of E_h_′ in the samples with a reference condition. One such suitable reference is that of complete reversible recovery after acute oxidative stress in a wild-type subject.

**Figure 4:**
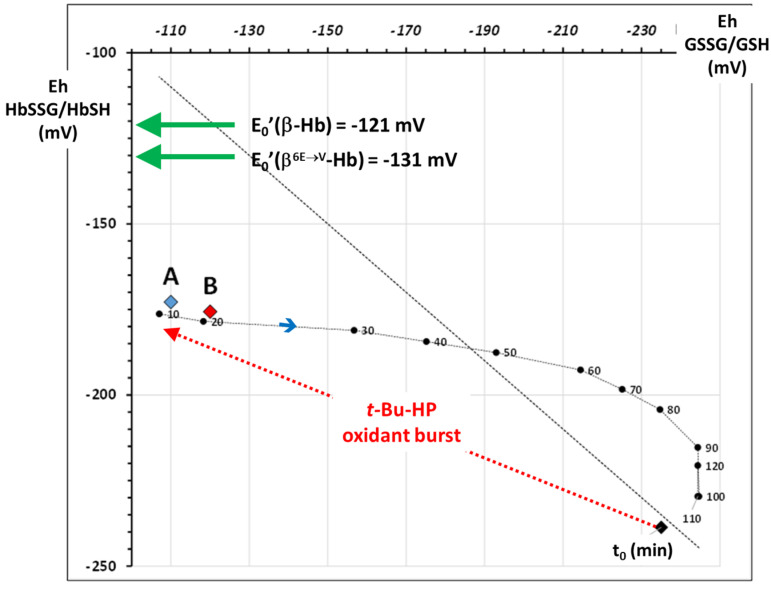
Plot of the E_h_ values of the glutathione (X-axis) and hemoglobin (Y-axis) redox pairs calculated from the oxidant challenge experiment carried out on wild-type, healthy RBCs [11,30]. T_0_ indicates the E_h_ before the oxidant challenge; black numbers indicate the E_h_ at progressive times during the recovery phase (from 10 min to 110 min). The two green arrows indicate the E_0_′ of the two hemoglobins studied in this report. The red and blue A and B diamonds show the values of E_h_ for HbSSG calculated for the two examined blood patients’ samples at admission and discharge, respectively.

This is indeed possible by referring again to the results of [15,31]. Briefly, we used [31] the original data of the previously published experiment [15] to calculate the E_h_‘ values of the two redox pairs, GSSG/GSH and HbSSG/HbSH, during the oxidant challenge and recovery phase, obtaining the time course of the experiment showed the displacement of the E_h_ potentials of both species from the “reduced” situation (equilibrium position close to the bisecting line) towards an oxidatively stressed one immediately after the addition of the oxidant and a gradual recovery of the conditions to the initial ones (Figure 4).

The same graph shows the E_h_′ values of the two samples A and B taken from the patient at admission (**A**, E_h_ = −172.8 ± 0.1 mV; red diamond) and at discharge (**B**, E_h_ = −175.7 ± 0.2 mV; blue diamond). These values describe levels of chronic oxidative stress comparable to those of the in vitro RBCs in the first 10–20 min after the acute oxidative stress and before starting the recovery phase. In addition, the difference of −2.8 ± 0.2 mV between the two samples may point to a slight decrease in the level of oxidative stress during the hospital stay. The E_h_ of the patient at discharge is not only statistically lower than the one at admission but is also comparable to the value calculated after the 20 min recovery in the “in vitro” experiment on oxidized RBC [30].

## 3. Discussion

We applied a previously developed method to assign a value to the hitherto unknown redox potential of the thiol group of the 93Cys residue in the β^6Glu→Val^ variant of the hemoglobin β-chain. The obtained value, more reducing (more negative) than that of the wild-type species, can explain the empirically observed higher level of glutathionylation in the RBCs of variant carriers [27].

It is of interest that the reactivity study of HbA and HbS towards the reference electrophilic reagent iodoacetic acid shows higher and faster alkylation of 93Cys in the wild-type than in the S-mutant form, both in the oxygenated and in the deoxy state [37]. This observation is a caveat towards adopting for proteins the straightforward correlation between a higher acidity and a more reducing potential that has been observed for small-molecule thiols [38].

### 3.1. The “Thermodynamic”—“Kinetic” Electrochemical Model of Thiol Oxidative Stress in RBCs

The use of redox potential calculated with the Nernst equation can improve the interpretation of cellular responses to oxidative stress [39,40,41].

From the simple side of data presentation, the redox potential E_h_ merges into a single calculated parameter of the two measured variables represented by the concentrations of the oxidized and reduced forms of a disulfide–thiol pair. This data transformation lowers the number of variables and simplifies data plotting.

In addition, from the side of the physical meaning of the measurements, the Nernstian potential of a disulfide–thiol pair depends not only on the concentration ratio but also on the total pool size of the species. This makes a difference from other redox systems, such as the NAD(P)H/NAD^+^ and FAD/FADH_2_, the E_h_ potential values of which only depend on the oxidized/reduced concentration ratio.

Furthermore, the relative values of the potentials calculated for different redox pairs, such as that of GSSG/GSH and that of Hb-SSG/Hb-SH, indicate the direction of the formal electron flux, as would the electromotive force of a reversible pile, where the lower-potential half-element “spontaneously donates” reducing equivalents to the higher-potential one. The use of these metrics requires the assessment of a fundamental constant for each species, the “normal” potential E_0_′, the value of which refers to standardized conditions of species concentration and specific conditions of temperature and pH for biologically relevant species.

The measurement of the E_0_′ of hemoglobin 93Cys, as well as the method that has been applied for the calculation, are thus embedded in a contemporary debate on the theoretically legitimate application of physico-chemical thermodynamic equilibrium models, such as the Nernstian electrochemical potential, to describe the biochemical phenomena inside cells and their substructures [41]. This highly conserved redox-active amino acid residue [42] plays a fundamental role in the preservation of the structural and functional integrity of hemoglobin in the oxidizing environment of the RBC [43,44].

This simplified approach considers the biochemical phenomena that occur inside physically segregated compartments, such as living cells, as being driven by thermodynamic-kinetic movers that operate under the laws of chemical equilibria in solution. This approach is particularly appealing in RBC biochemistry, due to the occurrence, in a deliberately [23,24,25,26,45] “simplified” model of RBC physiology, of the biochemical processes as they would in a conceivably “free solution state” of the RBC cytoplasm. This bio-organic reductionist paradigm is, in part, justified by the fact that the mature, circulating human RBCs do not have a nucleus, protein biosynthesis, or mitochondrial electron transport.

This trust in the paradigm had been pioneered by Schafer & Buettner [46] and by Jones and collaborators [40,47,48], who introduced the calculation of Nernstian potentials as a physically significant metrics for describing the relative levels of the free-thiol and disulfide forms of the soluble thiols in tissue, in the whole cells and in subcellular organelles in different stages of the response to physiological and stress-challenged conditions [39].

As an example of the application of redox potential metrics, one experiment measured the circadian changes in concentrations of plasma cystine/cysteine and glutathione disulfide/glutathione redox pairs in healthy volunteers [49] and calculated the respective E_h_ values from the measured concentrations. The time courses showed prominent and complex circadian variations, with E_h_ of the glutathione pair ranging from −118 to −123 mV, with maxima around 13:00 and 00:30 and minima reached around 08:00, 17:30, and 22:30, while that of the cysteine pair ranged from −73 to −79 mV, with the minimum reached at around 20:30 and the maxima at around 21:00 and 05:00. The minima for glutathione were shifted concerning those of cysteine that was introduced with the diet and was made available after food digestion.

A further example of the efficacy of the electrochemical equilibrium approach to highlight the in vitro resilience of RBCs to radical oxidant challenge has been reported in Section 2.3 above [15,31]. This experiment shows that the single oxidant burst did not irreversibly damage the redox homeostasis machinery of the healthy RBCs, which reacted to the oxidation of the intracellular glutathione and hemoglobin pools in a quantitatively reversible mode. The E_h_ metrics can thus be employed as a theoretically legitimate general frame to compare the effect of different conditions of oxidative stress, including the reversible physiological recovery phase, to an in vitro reference one. This is an appealing possibility in the dynamic study of oxidative stress phenomena occurring in vivo in patients and in subjects experiencing extreme environments.

However, in quantitative models of oxidative stress production and recovery, we should also consider the pivotal role of the enzymes that synthesize glutathione and recycle the disulfide-bound forms, as highlighted by Flohé [50,51]. His objection is, however, compatible with the results of the described oxidant challenge experiment, which measured resilience to a *single* oxidative exposure. It is reasonable to expect that *repeated* oxidative challenges without a suitable recovery phase, or *chronic* exposure to oxidizing conditions, will lead to a progressive inactivation of the redox enzymes [52,53]. A decrease in enzyme activity will, in turn, delay the recovery phase of the thiolome, causing high levels of the disulfide forms. This could be the case for several cohorts of chronically impaired subjects, such as heavy smokers [53], refractory overweight subjects [54], workers exposed to workplace pollutants [55], and patients with chronic respiratory impairment [32].

### 3.2. The S-Variant of Hemoglobin and Oxidative Stress in RBCs in Malaria Infection

The examined blood sample belongs to a subject whose heterozygous HbA+/HbS+ status is often associated with provenance from geographical regions with a high prevalence of malaria [56]. The evolutionary conservation of the heterozygous trait is due to the enhanced resistance of the carrier subjects to the effects of infection with *P. falciparum*, the causal agent of malaria [57,58]. According to abundant research, the peculiar physico-chemical properties of HbS make the cytoplasmic environment of the RBCs in the “sickled” shape less adapted to the growth of blood parasites, such as the developing forms of *P. falciparum*. Protein carbonylation is approximately fivefold higher in *P. falciparum* cultivated in heterozygous S-trait carriers than in wild-type RBCs [59]. The malaria parasite needs a specific redox environment to reside and multiply inside the RBCs [59] and challenges the pro-oxidant response of host RBCs with a unique form of “improved”, dimeric glutathione, trypanothione, that has better antioxidant properties than the ordinary monomeric tripeptide [60].

For this reason, while homozygous carriers of the sickle cell trait have poor chances of surviving in the reproductive age, heterozygous carriers, which express both the HbA and HbS, obtain an evolutionary advantage in geographical areas where malaria is endemic [57].

The redox balance of RBCs in subjects with SCD has been studied through several different indicators, which include the measurement of the total, reduced (thiol), and soluble disulfide form of glutathione [61]. The approx. 35% lower level of total soluble glutathione measured in SCD patients was explained as increased binding to proteins, although without explicit measurement of this compartment. An approximate calculation based on the reported information shows that this level may correspond to a total glutathionylated hemoglobin pool in the 7% range, a level comparable to the 5% measured as the sum of the glutathionylated forms in our examined patient (Table A3).

Glutathionylation is a protective process that modifies the conformation of the tetramer of Hb-S and hampers its polymerization, thus mitigating the “sickling” deformation of RBC shape and the cascade of pathological phenomena caused to the affected subject by this functional impairment [4,5,44,62].

Several biomarkers of oxidative stress, including the levels of reduced glutathione and enzyme activities, were measured in plasma and in RBC hemolysates of SCD patients treated or not with hydroxyurea, compared to controls, both in vivo and in vitro [63,64]. In vivo, hydroxyurea is a putative precursor of nitric oxide, the binding of which to circulating thiols triggers the formation of thiol disulfides. Treatment of RBCs with hydroxyurea triggers a dose-dependent increase in antioxidant defenses (increased activity of Superoxide Dismutase and Glutathione Peroxidase(s), decreased Glutathione Reductase(s) and free glutathione). Concomitantly, there is a switch of the glucose metabolism towards the production of reducing power through the activity increase in Hexokinase and Glucose-6-phosphate Dehydrogenase [64].

In the treatment of SCD, the failure of antioxidant treatments, such as administration of strongly reducing *N*-acetylcysteine (NAC) (E_0_ = −268 mV [25]) [65] to prevent occlusive crises, can be rationalized as the consequence of an excessive imbalance of the redox equilibrium towards reduction, with decrease in the protective glutathionylated form of HbS and enhanced formation of intra-erythrocyte hemoglobin fibrils. Concerning the use of antioxidant therapy for SCD treatment, de Silva et al. [66] call for the use of NAC and Melatonin in combination. A recent study [67] measured oxidative stress in the RBCs of wild-type Hb young obese subjects undergoing a weight-loss protocol that included supplementation with melatonin. Contrary to expectations, the melatonin-treated arm showed enhanced rather than lowered levels of oxidative stress, including HbSSG. Understanding how and if this observation can be useful for SCD management calls for additional studies.

In conclusion, the availability of the E_0_′ redox potentials of the β-93Cys thiolate group of hemoglobin variants could be a useful tool that may add to the limited panel of oxidative stress biomarkers of RBC. The use of the E_h_ redox potential in addition to the simple % of HbSSG may more efficiently assess the effect of pharmacological and nutraceutical interventions [68,69,70].

## 4. Materials and Methods

### 4.1. Patient History

The 41-year-old adult male subject to whom the blood samples belong was born in Cameroun. He had been admitted recently to the hospital for treatment of pneumonia and had been enrolled in a wide, approved research protocol aimed at studying biomarkers of hypoxia. Among the research biomarkers of the study, the measurement of glutathionyl–hemoglobin was included as an indicator of oxidative stress.

### 4.2. Blood Samples

Two blood samples were obtained, one at admission to and one at discharge from the hospital, from the healed patient. Pre-analytical blood treatment (coding, fractionation, and storage) was performed at the times of admission and discharge, and routine blood analyses were executed by the clinical laboratory. The measurement of glutathionyl–hemoglobin was performed after the end of all patients’ enrolment and discharge. Therefore, the condition of being an otherwise healthy carrier of the HbS hemoglobin variant became known to the investigators only during the post-acquisition review of the mass spectra, when the measurement of glutathionyl–hemoglobin was performed in the study samples batch.

### 4.3. Measurement of Glutathionyl–Hemoglobin

The measurement of glutathionyl–hemoglobin in the patient’s blood sample was performed during a routine research measurement that employed the published method [71]. Briefly, a micro-sample of 10 mM titrated hemolysate was measured by Matrix-Assisted Laser Desorption mass spectrometry in a Time-of-Flight instrument (MALDI-ToF) in a Bruker Autoflex II instrument (Bruker, Inc., Bremen, Germany).

Sinapinic acid (SA; 10 mM dissolved in 0.1% trifluoroacetic acid-acetonitrile) was used as MALDI matrix, in a 1:10,000 molar proportion to the hemolysate. An automated procedure was used for laser firing, and the sample was measured in quadruplicate.

### 4.4. Calculation of the Molecular Masses of Hemoglobin Proteins and Variants

The amino acid sequences of the wild-type and variant hemoglobin chains were obtained from the UniProt database (https://www.uniprot.org/uniprotkb/P68871/entry#structure, accessed on 4 November 2025); their elemental compositions and those of the post-translational modifications (glycation and glutathionylation) were calculated with a custom spreadsheet. The corresponding molecular masses and isotope pattern profiles were calculated from the elemental composition with the Envipat calculator (https://www.envipat.eawag.ch/index.php, accessed on 4 November 2025) [72], at a value of mass resolution (M/ΔM) of 1000, to yield the results of Table A1. All results were downloaded to Excel spreadsheets for further elaboration.

### 4.5. Data Analysis

An original, custom spreadsheet was employed to analyze and integrate the profile mass spectra, extracted as raw, externally calibrated .txt files from the instrument analysis files. The area integration routine was modified in the spreadsheet to include the signals belonging to the unexpected protein species. The obtained *m*/*z* values (MH^+^ species) are reported in Table A1, as the mean and interval of the four separate determinations.

A specific worksheet was prepared to perform the calculations for the estimation of E_0_ from the raw data.

### 4.6. Calculation of the E_0_ Electrochemical Potential of the HbS Glutathionyl–Hemoglobin

The calculation of the E_0_′ electrochemical potential of the unexpected protein species was carried out by adopting the “equilibrium model”, according to the published method [31] employed to calculate the E_0_′ of the wild-type glutathionyl–hemoglobin in an ex vivo, in vitro oxidant challenge experiment performed on human RBCs [15].

Briefly, it is assumed that, in the measured sample, the relative concentrations of “reduced” (thiol-free) and “oxidized” (mixed-disulfide) forms of the glutathionyl–hemoglobins correspond to those derived from the Nernst equation for the electrochemical cell pair of Equation (1)

β-Hb-SSG/β-Hb-SH//β^6Glu→Val^ Hb-SSG/β^6Glu→Val^ Hb-SH (1)

for which the redox equilibrium condition (E_eq_) is calculated from Equation (2)

E_eq_ = E_0_(β-Hb) + RT/nF ln [β-Hb-SSG]/[β-Hb-SH]^2^ =
=E_0_β^6Glu→Val^ Hb) + RT/nF ln [β^6Glu→Val^ Hb-SSG]/[β^6Glu→Val^ Hb-SH] (2)

The value for E_0_′(β-Hb) is −121 mV [31].

The calculation of actual intra-erythrocyte concentrations of the proteins in Equation (2) is performed by referring to their relative proportions (Table A2) to the total hemoglobin concentration, which is calculated from the hematocrit and total Hb concentration according to Equation (3).

Hb_RBC_ (mM) = 10^3^*(10*Hb_WB_ (g/dL)/64.454)/Ht% (3)

## 5. Conclusions

This is the first attempt to assign a value to the redox potential of the thiol group in the β-93Cys residue of the point-mutated β^6Glu→Val^ isoform of human hemoglobin, responsible for the sickle cell trait. The obtained value confirms that the thiol group is more reducing and therefore more susceptible to oxidation, nucleophilic attack, and thiol–disulfide exchange in the mutation-carrying than in the wild-type chain (E_0_′ of −131 mV vs. −121 mV). This feature explains why HbS is more prone to glutathionylation.

The assessment of the redox status of RBCs by the stable biomarker HbSSG% and calculation of HbSSG E_h_ could improve the understanding of oxidative stress and help the rational treatment with diets and nutraceutical supplements.

## Data Availability

The original contributions presented in this study are included in the article/Supplementary Materials. Further inquiries can be directed to the corresponding author.

## Supplementary Materials

The following supporting information can be downloaded at: https://www.mdpi.com/article/10.3390/molecules30224342/s1. Figure S1. Main processes of oxidative stress in RBCs through the generation of hydrogen peroxide and organic hydroperoxides, and coping mechanisms by glutathione and hemoglobin; Figure S2. Main processes of restoration of the reduced thiol pool in RBCs. Refs. [73,74,75] are cited in Supplementary Materials.

## Abbreviations

The following abbreviations are used in this manuscript:

βHb	wild-type (6Glu) β-chain of human hemoglobin
β^6Glu→Val^ Hb	6Glu > Val point-mutated β-chain of human hemoglobin
E_0_′	Standard electrochemical reduction potential (at “near-physiological” pH 7)
Eh	Concentration-dependent electrochemical reduction potential
GSH	Glutathione, “reduced” thiol form
GSSG	Glutathione, “oxidized” disulfide form
Prot-SSG	Glutathionylated form of generic protein
HbA	Main wild-type form of adult human hemoglobin α_2_β_2_
HbS	Main mutated form of sickle cell hemoglobin α_2_(β^6E→V^)_2_
HbA-SH	Main wild-type form of adult human hemoglobin α_2_β_2_ thiol form
HbS-SH	Main mutated form of sickle cell hemoglobin α_2_(β^6E→V^)_2_ thiol form
HbA-SSG	Glutathionyl–hemoglobin A, glutathionylated at β-93Cys
HbS-SSG	Glutathionyl–hemoglobin S, glutathionylated at β-93Cys
HbA_1c_	Glycated hemoglobin A
β-HbGlc	Glycated hemoglobin A (β-chain)
β^6Glu→Val^ HbGlc	Glycated hemoglobin S
RBC	Red blood cell(s)
SCD	Sickle Cell Disease

## Appendix A. Characterization of the Main Proteins in the RBC Samples by MALDI-ToF Mass Spectrometry

The main proteins in the RBC samples were identified by matching the *m*/*z* values measured in the internally calibrated mass spectra to those calculated from the respective amino acid sequences and putatively occurring post-translational modifications (PTM), as summarized in Table A1. Signal intensities were obtained with the spreadsheet described in Ref. [71].

**Table A1:** Summary of the hemoglobin species identified in the mass spectra of the two blood samples from the HbS carrying patient.

				MH^+^	Samp_1		Samp_2	
Type	Protein	PTM ^1^	Molecular Formula	*m*/*z* calc	*m*/*z*	Δ_*m*/*z* ^2^	*m*/*z*	Δ_*m*/*z* ^2^
wt	α-Hb	-	C685 H1071 N187 O194 S3	15,127.5	15,127.4	0.4	15,127.0	0.9
wt	α-Hb	Glc	C691 H1081 N187 O199 S3	15,289.7	15,289.1	0.5	15,288.5	1.6
wt	β-Hb	-	C724 H1119 N195 O201 S3	15,868.4	15,868.2	0.5	15,867.9	0.7
wt	β-Hb	Glc	C730 H1129 N195 O206 S3	16,030.5	16,044.1	3.8	16,043.8	1.8
wt	β-Hb	SSG	C734 H1134 N198 O207 S4	16,173.6	16,172.9	0.6	16,173.0	6.0
β^6Glu→Val^	β-Hb	-	C724 H1121 N195 O199 S3	15,838.4	15,838.4	0.5	15,838.2	0.8
β^6Glu→Val^	β-Hb	Glc	C730 H1131 N195 O204 S3	16,000.5	15,999.5	1.4	15,999.1	6.4
β^6Glu→Val^	β-Hb	SSG	C734 H1136 N198 O205 S4	16,143.7	16,143.4	2.6	16,141.0	3.0

^1^ Post Translational Modification. ^2^ Max-min interval of centroid *m*/*z*, to be compared to the width of the isotope envelope at 1000 resolution, of approx. 14 Da.

**Table A2:** Relative proportion (%) of β-Hb and β^6Glu→Val^ Hb in the two blood samples from the HbS carrying patient ^1^.

Repl	βHb ^1^	β^6Glu→Val^ βHb ^1^	βHb ^1^	β^6Glu→Val^ βHb ^1^
	Samp1		Samp2	
Repl_1	61.1	38.9	61.2	38.8
Repl_2	60.3	39.7	60.6	39.4
Repl_3	60.2	39.8	61.6	38.4
Repl_4	60.4	39.6	61.1	38.9
***M***	**60.5**	**39.5**	**61.2**	**38.8**
DS	**0.3**	**0.3**	**0.3**	**0.3**
CV%	**0.7**	**1.1**	**0.6**	**1.0**

^1^ Integration from custom spreadsheet, only the species at *m*/*z* 15,868.4 (β-Hb) and 15,838.4 (β^6Glu→Val^ Hb), respectively.

**Table A3:** Proportions (%) of the major and post-translationally modified forms of βHb and β^6Glu→Val^ Hb in the two blood samples from the HbS—carrying patient ^1^.

		Samp	A					Samp	B			
	Replicates						Replicates					
Species	1	2	3	4	M	SD	1	2	3	4	M	SD
β-HbSH	55.8	55.6	54.9	55.5	**55.5**	**0.4**	56.6	56.1	56.4	56.4	**56.4**	**0.2**
β-HbGlc	3.7	3.2	3.6	3.3	**3.4**	**0.2**	3.3	3.3	4.2	3.6	**3.6**	**0.4**
β-HbSSG	2.7	2.7	2.7	2.7	**2.7**	**0.0**	2.2	2.2	2.2	2.2	**2.2**	**0.0**
β^6Glu→Val^-HbSH	35.5	36.6	36.3	36.4	**36.2**	**0.5**	35.8	36.4	35.2	35.9	**35.8**	**0.5**
β^6Glu→Val^-HbGlc	<0.5	<0.5	<0.5	<0.5			<0.5	<0.5	<0.5	<0.5		
β^6Glu→Val^-HbSSG	2.3	1.9	2.5	2.1	**2.2**	**0.3**	2.1	2.1	2.0	2.0	**2.0**	**0.0**
∑ all HbSSG					**4.9**	**0.2**					**4.2**	**0.0**

^1^ Integration from custom spreadsheet, sum of all considered species, α-Hb excluded.

## Appendix B. Calculation of the E_0_ of S-Hb with the Equilibrium Method

The calculation with the equilibrium method is reported in Table A4 below and follows the method described in ref. [31], separately for the two blood samples A (at admission) and B (at healed discharge).

**Table A4:** Development of the calculation to estimate the value of E_0_′ for the thiol group of the Hb-S variant of hemoglobin from the composition of the protein forms in the two blood samples (A and B) from the β^6E→V^ Hb-S—carrying patient.

**Samp**				**A**						**B**			
**Hb (g/dL)** ^a^				11.9						13.9			
**Ht %** ^a^				35.6%						42.6%			
**[Hb] mM**				5.02						4.90			
**Species**		**1**	**2**	**3**	**4**	**M**	**SD**	**1**	**2**	**3**	**4**	**M**	**SD**
β-HbA-SH		55.8%	55.6%	54.9%	55.5%	***55.5%***	***0.4%***	56.6%	56.1%	56.4%	56.4%	***56.4%***	***0.2%***
β-HbGlc		3.7%	3.2%	3.6%	3.3%	***3.4%***	***0.2%***	3.3%	3.3%	4.2%	3.6%	***3.6%***	***0.4%***
β-HbSSG		2.7%	2.7%	2.7%	2.7%	***2.7%***	***0.0%***	2.2%	2.2%	2.2%	2.2%	***2.2%***	***0.0%***
β^6Glu→Val^ HbA-SH		35.5%	36.6%	36.3%	36.4%	***36.2%***	***0.5%***	35.8%	36.4%	35.2%	35.9%	***35.8%***	***0.5%***
β^6Glu→Val^ HbGlc		<0.5%	<0.5%	<0.5%	<0.5%			<0.5%	<0.5%	<0.5%	<0.5%		
β^6Glu→Val^ HbS-SSG		2.3%	1.9%	2.5%	2.1%	***2.2%***	***0.3%***	2.1%	2.1%	2.0%	2.0%	***2.0%***	***0.05%***
β-HbSH	mM	2.80	2.79	2.76	2.78			2.77	2.75	2.76	2.76		
β-HbGlc	mM	0.18	0.16	0.18	0.17			0.16	0.16	0.21	0.17		
β-HbSSG	mM	0.14	0.14	0.13	0.14			0.11	0.11	0.11	0.11		
β^6Glu→Val^-HbSH	mM	1.78	1.84	1.82	1.83			1.75	1.78	1.72	1.76		
β^6Glu→Val^-HbGlc	mM												
β^6Glu→Val^ HbSSG	mM	0.12	0.10	0.13	0.11			0.10	0.10	0.10	0.10		
***Nernst calculation***													
RT/nF (mV)		29.5	29.5	29.5	29.5			29.5	29.5	29.5	29.5		
E_0__β-Hb (mV)	mV	−121	−121	−121	−121			−121	−121	−121	−121		
β _[OX]/[RED]^2		0.0174	0.0174	0.0177	0.0175			0.0139	0.0143	0.0142	0.0138		
β^6Glu→Val^ [OX]/[RED]^2		0.0372	0.0284	0.0378	0.0316			0.0333	0.0319	0.0331	0.0316		
β_logRat		*−1.758*	*−1.760*	*−1.752*	*−1.758*			*−1.857*	*−1.846*	*−1.848*	*−1.860*		
β^6Glu→Val^ _logRat		*−1.430*	*−1.547*	*−1.422*	*−1.501*			*−1.477*	*−1.496*	*−1.480*	*−1.501*		
A_E_h_ (mV)		−173	−173	−173	−173	***−172.8***	***±0.1***	−176	−175	−176	−176	***−175.7***	***±0.2***
β^6Glu→Val^ _E_h_-E_0_ (mV)		−42	−46	−42	−44	−43.5	±1.8	−44	−44	−44	−44	−43.9	***±***0.3
E_0_′β^6Glu→Val^-Hb (mV)	mV	−130.7	−127.3	−130.7	−128.6	***−129.3***	***±1.7***	−132.2	−131.3	−131.9	−131.6	***−131.7***	***±0.4***

^a^ Data from the clinical laboratory.

The estimated mean E_0_′ value resulting from the calculation for the disulfide–thiol redox process of the β^6Glu→Val^-93Cys thiol group is thus −130.5 ± 1.7 mV.
