# Supplementary material for: Redox Potential (E0′) of the β-Chain 93Cys of HbS Measured with the Equilibrium Technique in a Heterozygous Sickle Cell Carrier Subject
Source: Molecules. 2025 Nov 10;30(22):4342. doi: 10.3390/molecules30224342 (PMC12655699; doi:10.3390/molecules30224342)
Supplement: Supplementary file 1 [file molecules-30-04342-s001.zip › molecules-3954260-supplementary.pdf]

Redox potential (E_0') of the β-chain 93Cys of HbS measured with the equilibrium technique in a heterozygous sickle cell carrier subject

Federico Maria Rubino [1]*, Aldijana Sadikovic[1], Camillo Morano[1], Michele Dei Cas[1], Monica Bignotto[1], Sara Ottolenghi[2], Michele Mondoni[3], Davide Chiumello[4], Michele Samaja[1], Rita Paroni[1]

Supplementary information

A few important biochemical processes of intracellular oxidative stress and coping mechanisms in RBCs are outlined in the scheme of Figure S1.

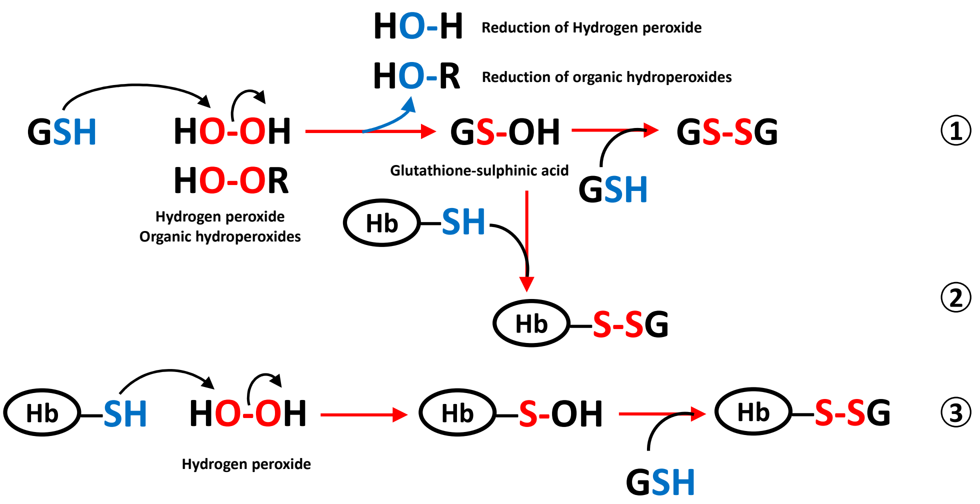

Figure S1. Main processes of oxidative stress in RBCs through the generation of hydrogen peroxide and organic hydroperoxides, and coping mechanisms by glutathione and hemoglobin. (1) Quenching of H_2O_2 and hydroperoxides by glutathione oxidation through sulphinic acid (GS-OH) to glutathione disulfide; (2) quenching of sulphinic acid labile intermediate by reversible glutathionylation of hemoglobin; (3) quenching of H_2O_2 by the Cys^{93} thiol group of hemoglobin, formation of sulphinic acid, and reaction with glutathione.

The sulphinic acid intermediate (Cy-S-OH), directly generated from thiol groups by reaction with hydrogen peroxide or by organic peroxides [1], is a key player in the complex network of oxidation stress events. The other component can be another molecule of glutathione, to generate glutathione disulfide (GSSG), or the cysteine residue of proteins, to generate glutathione "mixed disulfide" forms (Prot-SSG).

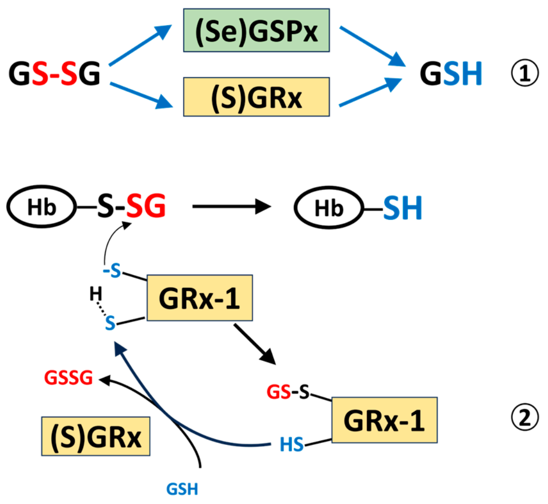

Figure S2. Main processes of restoration of the reduced thiol pool in RBCs. (1) glutathione disulfide reduction by Se-glutathione peroxidase and NADPH-glutathione reductase; (2) glutathione-hemoglobin reduction by reversible glutathionylation of GRx-1.

Figure legend.

GSH: glutathione

GS-SG: glutathione disulfide

Hb-SH: hemoglobin

Hb-S-SG: glutathionylated hemoglobin

(Se)GSPx: glutathione:hydrogen-peroxide oxidoreductase (EC 1.11.1.9), Selenium dependent

(S)GRx: Glutathione reductase (EC 1.8.1.7), NADH-FAD dependent

GRx-1: Glutaredoxin-1 (EC 1.8.1.8) protein disulfide reductase

The restoration of the reduced thiol pool in the RBCs includes two main multi-enzymatic mechanisms of glutathione disulfide reduction [2] and at least one for direct reduction of glutathionyl-hemoglobin [3,4,5,6], as outlined in the scheme of Figure S2.

References

1. Regazzoni, L.; Panusa, A.; Yeum, K.-J.; Carini, M.; Aldini, G. Hemoglobin Glutathionylation Can Occur Through Cysteine Sulfenic Acid Intermediate: Electrospray Ionization LTQ-Orbitrap Hybrid Mass Spectrometry Studies. J. Chromatogr. B 2009, 877, 3456–3461.
2. Deponte, M. Glutathione catalysis and the reaction mechanisms of glutathione-dependent enzymes. Biochim. Biophys. Acta 2013, 1830, 3217–3266
3. John J. Mieyal, David W. Starke, Stephen A. Gravina, Chantal Dothey, James S. Chung. Thioltransferase in Human Red Blood Cells: Purification and Properties. Biochemistry, 30, 6088-6097 (1991)
4. Gravina, S.A., Mieyal, J.J. Thioltransferase is a specific glutathionyl mixed disulfide oxidoreductase. Biochemistry, 32 (1993), pp. 3368-3376
5. J.J. Mieyal, M.M. Gallogly, S. Qanungo, E.A. Sabens, M.D. Shelton. Molecular mechanisms and clinical implications of reversible protein S-glutathionylation. Antioxidants Redox Signal., 10 (2008), pp. 1941-1988
6. Lang L, Reinert P, Diaz C, Deponte M. The dithiol mechanism of class I glutaredoxins promotes specificity for glutathione as a reducing agent. Redox Biol. 2024 Dec;78:103410. doi: 10.1016/j.redox.2024.103410. PMID: 39488995
